# First evidence of enterovirus A71 and echovirus 30 in Uruguay and genetic relationship with strains circulating in the South American region

**DOI:** 10.1371/journal.pone.0255846

**Published:** 2021-08-12

**Authors:** Andrés Lizasoain, Daiana Mir, Matías Salvo, Viviana Bortagaray, Gisela Masachessi, Adrián Farías, Nélida Rodríguez-Osorio, Silvia Nates, Matías Victoria, Rodney Colina

**Affiliations:** 1 Laboratorio de Virología Molecular, Departamento de Ciencias Biológicas, Centro Universitario Regional Litoral Norte, Universidad de la República, Salto, Uruguay; 2 Unidad de Genómica y Bioinformática, Departamento de Ciencias Biológicas, Centro Universitario Regional Litoral Norte, Universidad de la República, Salto, Uruguay; 3 Laboratorio de Gastroenteritis Virales y Sarampión, Instituto de Virología Dr. J. M. Vanella, Facultad de Ciencias Médicas, Universidad Nacional de Córdoba, Córdoba, Argentina; Taif University, SAUDI ARABIA

## Abstract

Human enteroviruses (EVs) comprise more than 100 types of coxsackievirus, echovirus, poliovirus and numbered enteroviruses, which are mainly transmitted by the faecal-oral route leading to diverse diseases such as aseptic meningitis, encephalitis, and acute flaccid paralysis, among others. Since enteroviruses are excreted in faeces, wastewater-based epidemiology approaches are useful to describe EV diversity in a community. In Uruguay, knowledge about enteroviruses is extremely limited. This study assessed the diversity of enteroviruses through Illumina next-generation sequencing of VP1-amplicons obtained by RT-PCR directly applied to viral concentrates of 84 wastewater samples collected in Uruguay during 2011–2012 and 2017–2018. Fifty out of the 84 samples were positive for enteroviruses. There were detected 27 different types belonging to *Enterovirus A* species (CVA2-A6, A10, A16, EV-A71, A90), *Enterovirus B* species (CVA9, B1-B5, E1, E6, E11, E14, E21, E30) and *Enterovirus C* species (CVA1, A13, A19, A22, A24, EV-C99). Enterovirus A71 (EV-A71) and echovirus 30 (E30) strains were studied more in depth through phylogenetic analysis, together with some strains previously detected by us in Argentina. Results unveiled that EV-A71 sub-genogroup C2 circulates in both countries at least since 2011–2012, and that the C1-like emerging variant recently entered in Argentina. We also confirmed the circulation of echovirus 30 genotypes E and F in Argentina, and reported the detection of genotype E in Uruguay. To the best of our knowledge this is the first report of the EV-A71 C1-like emerging variant in South-America, and the first report of EV-A71 and E30 in Uruguay.

## Introduction

Human enteroviruses (EVs) are ubiquitous viruses mainly transmitted among all age groups in a community by the faecal-oral route [1]. They are associated with a large number of clinical conditions, ranging from nonspecific symptoms as fever and malaise, mild conditions as herpangina, gastroenteritis or hand-foot-and-mouth disease (HFMD), up to severe diseases as aseptic meningitis (AM), encephalitis, pancreatitis, myocarditis, or acute flaccid paralysis (AFP), among others [2].

EVs include around 100 types of coxsackievirus, echovirus, poliovirus and numbered enteroviruses, which are genetically classified inside four viral species (*Enterovirus A* to *Enterovirus D*) in the genus *Enterovirus* from the family *Picornaviridae*. *Enterovirus A* species includes several coxsackievirus type A (CVA2-A8, A10, A12, A14-A16) and numbered enterovirus A71, A76, A89-A91, A114, A119-A121. *Enterovirus B* species includes all the coxsackievirus B types (B1-B6), all the echovirus types (E1-E7, E9, E11-E21, E24-E27, E29-E33), a coxsackievirus A9 type, and several numbered enteroviruses (EV-B69, EV-B73-B75, EV-B77-B88, EV-B93, EV-B97, EV-B98, EV-B100, EV-B101, EV-B106, EV-B107, and EV-B111). *Enterovirus C* species includes the three poliovirus types (PV1-PV3), several coxsackievirus A types (CVA1, A11, A13, A17, A19-A22, A24), and numbered enterovirus C95, C96, C99, C102, C104, C105, C109, C113, and C116-C118. *Enterovirus D* species includes a few numbered enteroviruses named D68, D70, D94, and D111 [3].

The epidemiology of EVs is a dynamic phenomenon that deserves continuous surveillance since novel EV types are frequently discovered, and types originally associated with a specific disease, could emerge associated with novel conditions [4–9]. During the last decades, poliovirus has been a priority for public health surveillance systems worldwide due to its high incidence in AFP cases [10]. In the poliovirus eradication era, other non-polio EVs are being studied for their role as emerging pathogens associated with AFP, or as causing agents of central nervous system (CNS) disease outbreaks [10–13]. Human enterovirus A71 (EV-A71) has been historically associated with HFMD, including severe forms of the disease, and also is strongly associated with severe and sometimes fatal CNS infections. Therefore, EV-A71 is currently considered as an emerging EV with serious consequences for human health. Several molecular epidemiology and vaccine development efforts are on the way for its containment and management. In fact, since 2015–2016 there are three inactivated, whole-virus EV-A71 vaccines licensed by China National Medical Products Administration (NMPA), which are currently commercially available. The vaccines were formulated with the most prevalent EV-A71 lineage in China as virus seed (C4 lineage), and elicited cross-protection against prominent epidemic lineages reported worldwide over the past decade [14–16].

On the other hand, echovirus 30 (E30), an *Enterovirus B* species member, is frequently associated with neurological symptoms, mainly AM [17, 18]. Its presence among a population seems to be cyclic, characterised by repeated epidemics -frequently over large geographic areas- every 3 to 5 years [19, 20].

Wastewater samples contain a high concentration of EVs particles as a result of their excretion in human faeces during the infection by distinct types. Wastewater Based Epidemiology (WBE) approaches are useful for describing the molecular diversity of EVs either as a complement of clinical surveillance, or as a tool to obtain information regarding enteroviruses when clinical surveillance is not done in a community. WBE has displayed a valuable role both in developed and developing areas of the world, increasing our knowledge about circulation of many enteroviruses, including emerging variants of public health concern [21–23].

Most infections by EVs are asymptomatic, which could lead to a silent circulation of many types in a community until the occurrence of outbreaks of diverse diseases [24–26]. Therefore, the possibility of detecting emerging types in wastewater, before the appearance of clinical cases, makes WBE of EVs, a fundamental tool for epidemiological surveillance.

South America is often described as a region with scarce available genetic information regarding EVs [20, 27]. Particularly in Uruguay, knowledge about molecular diversity of circulating EVs is limited [28]. To assess the diversity of circulating EVs types in the Uruguayan population, we performed an amplicon-deep sequencing approach of Uruguayan wastewater samples collected in two different sampling periods in four cities (Bella Unión, Salto, Paysandú, and Fray Bentos). Additionally, as part of a project to characterize the diversity of EVs in the South American region, two EV types detected in this project (EV-A71 and E30) were subjected to phylogenetic analysis along with sequences of strains previously detected in wastewater from Córdoba, Argentina [29], to evaluate viral dynamics within a regional context and to identify viral introductions and country-specific transmission clusters.

## Materials and methods

### Wastewater specimens

**Fig 1:**
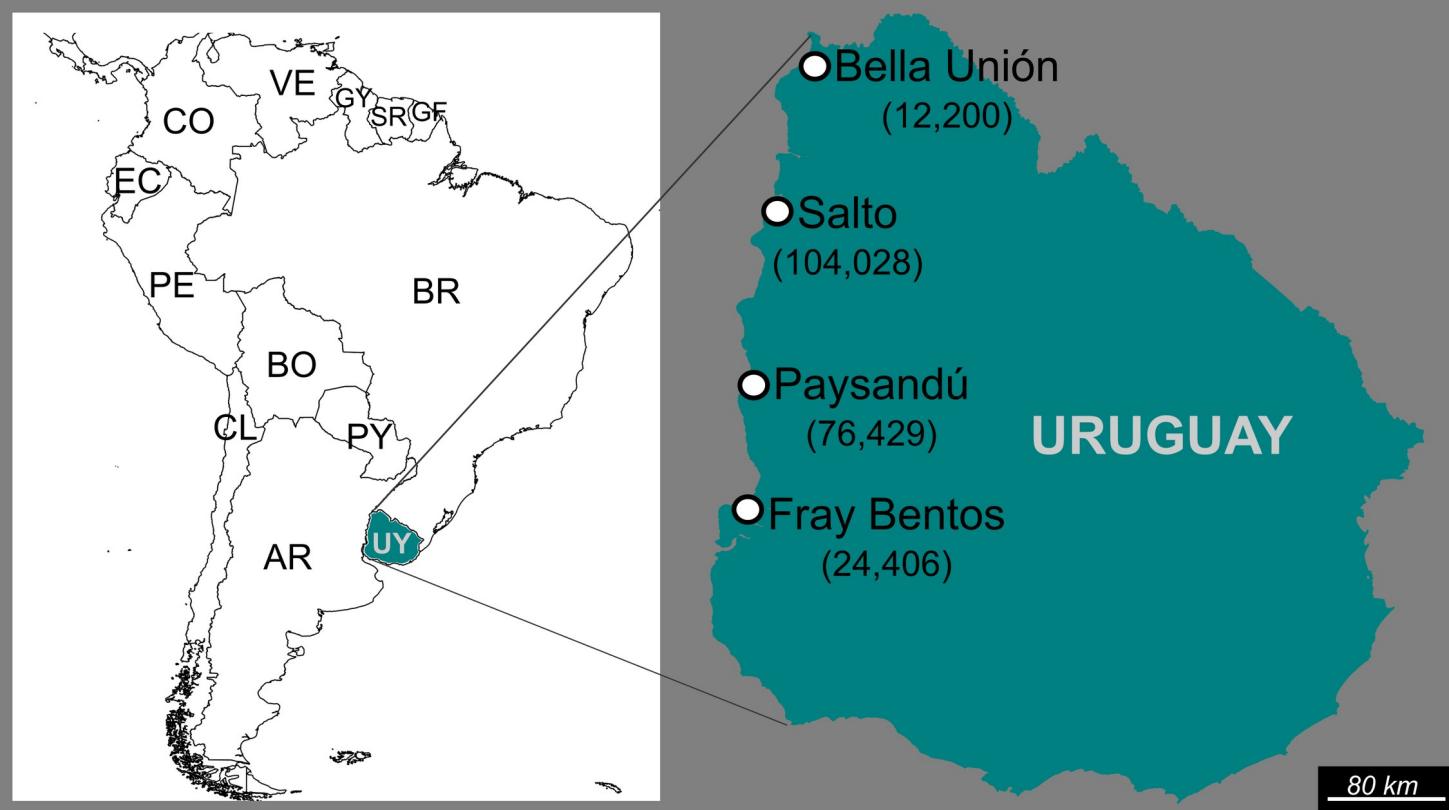
Geographic location of the four Uruguayan cities in which wastewater samples for this study were collected. In the left panel, a map of South America shows Uruguay in green. In the right panel, a zoom-view of the map of Uruguay shows Bella Unión, Salto, Paysandú, and Fray Bentos locations. Population size of each city is also indicated. South American countries are named according to the Alpha-2 code as described in the ISO 3166 international standard.

This work was based on the study of two sets of wastewater specimens collected during different periods in cities from the North-western region of Uruguay (Fig 1). SET_1 comprises specimens (100 ml each) collected monthly between March 2011 and February 2012 in four Uruguayan cities: Bella Unión (30°15’59.55’’S, 57°36’4.79’’W), Salto (31°23’18.82’’S, 57°58’35.09’’W), Paysandú (32°19’38.38’’S, 58°6’3.83’’W) and Fray Bentos (33°7’8.95’’S, 58°20’3.38’’W). SET_2 comprises specimens (100 ml each) from three of the previous four cities (Bella Unión, Salto and Fray Bentos) also collected once a month between March 2017 and February 2018 at the same sampling points from 2011–2012. Specimens from Salto, Paysandú and Fray Bentos consist of domestic raw wastewater, collected from master-pipes before discharging onto the Uruguay river. Nevertheless, the specimens from Bella Unión were collected from a natural glen that receives the effluent from a wastewater stabilization-pond.

One sampling point was determined in each city and since all of them were of public access, none permits were required for collecting.

Samples (100 ml) were concentrated as previously described in Lizasoain et al. (2018) [28] using a method of adsorption-elution to a negatively charged membrane and concentrating virus in 1 ml of final eluate per sample (100-fold concentration).

### Human enterovirus detection, next-generation sequencing and bioinformatics

Viral genomes were extracted from 140 μL of viral concentrate using the QIAamp® Viral RNA Mini Kit (Qiagen™, Hilden, Germany) according to manufacturer’s instructions. A RT-nested PCR method proposed by Nix et al. [30] was used to amplify a partial segment of VP1-coding segment with primers AN32-AN35 in the reverse transcription, and primers 222/224 and AN88/AN89 in first and second PCR rounds, respectively. At the second round, AN88 and AN89 primers were modified by addition of Illumina Universal Adapter sequences at the 5´ends, according to the protocol of library preparation for metagenomic sequencing [31] in Illumina MiSeq platform (Illumina Inc., San Diego, CA, USA). PCR products were loaded into agarose gels and after the electrophoresis, bands of the expected size were excised and purified with the PureLink® Quick Gel Extraction and PCR Purification Combo Kit (Invitrogen-Life Technologies, Carlsbad, CA, USA). Macrogen Inc. Next Generation Sequencing Service (Seoul, Republic of Korea), barcoded each sample and prepared the libraries with Nextera XT Index Kit (Illumina Inc., San Diego, CA, USA). Samples were sequenced on Illumina MiSeq 2 × 300 bp, producing paired end reads. Libraries from 2011–2012 and 2017–2018 were sequenced in two independent runs.

Raw Illumina reads were paired using merge pairs algorithm with a minimum overlap length of 50 bp in VSEARCH v2.11 [32]. The resulting contigs (merged paired reads) were filtered out if their lengths were <100 bp, and if they contained homopolymer tracks >8 bp in length. Quality trimming of contigs was performed by using—fastq_filter command and contigs with more than 1.0 total expected errors (—fastq_maxee 1.0) were discarded. In order to save time in further analyses, a dereplication step was performed using VSEARCH—derep_fulllength algorithm on the processed contigs to find unique sequences by clustering at 100% sequence identity. All unique sequences without duplicates (singletons) were removed since they were considered probable sequencing-error products. Probable chimeras (abskew = 2) were also removed from each sample.

Clusters of contigs were generated with VSEARCH using identity criterion of 97%, adopting a representative sequence (centroid sequence) of each cluster for further analyses. Finally, centroids sequences were mapped by VSEARCH’s—usearch_global at 80% sequence identity against a Customized Human Enteroviruses Database (CHED v1.0), composed of 30,850 sequences of the viral capsid VP1 protein-coding region from 111 enterovirus types, which were downloaded from NCBI nucleotide database [33].

### Phylogenetic characterisation of enterovirus A71 and echovirus 30

All EV-A71 and E30 VP1 gene sequences > 800 nt with known location and sampling date available in the GenBank by September 4^th^, 2020 were downloaded and combined with: 1) strains reported in this study and their best BLAST hits; 2) partial VP1 sequences from Córdoba city (Argentina) previously described by our group (13 sequences of EV-A71 and 20 sequences of E30), and their best BLAST hits.

Some reference sequences for EV-A71 and E30 used by Bessaud et al. [34] and Lema et al. [35] respectively, were included in both datasets. This resulted in a final dataset composed by 7,658 sequences of EV-A71 and a dataset of 1,297 E30 sequences. Both datasets were aligned with the Muscle program [36]. Maximum likelihood (ML) phylogenetic trees were inferred with FastTree v2.1 software [37] under the GTR+Gamma20 model. The reliability of the phylogenies was estimated with the approximate likelihood-ratio test (aLRT) based on a Shimodaira–Hasegawa-like procedure [38, 39]. Phylogenetic trees were visualized and edited with FigTree v1.4.3.

Nucleotide sequences of EV-A71 and E30 from Uruguay reported in this study were deposited in GenBank and are available with the accession numbers MW196710-MW196712, MW196730-MW196732 and MW196734.

## Results

### Human enterovirus detection and characterization

Fifty out of a total of 84 Uruguayan wastewater samples collected along 2011–2012 and 2017–2018, were positive for EVs (Table 1).

**Table 1:** Human enterovirus positivity by city and next-generation sequencing performance of VP1-amplicons from Uruguayan wastewater samples.

			Reads^a^					
	n^c^	pos^d^/n	Paired raw	Pair merged	After quality filters	Mapped	EV types	OTUs^e^
**2011–2012**								
**BU** ^**b**^	12	2/12	123,658	71,433	66,894	81	2	2
			±30,442	±24,204	±24,620	±112		
**SA** ^**b**^	12	9/12	141,316	108,839	102,456	51,495	9	20
			±11,113	±8,792	±8,258	±37,689		
**PY** ^**b**^	12	6/12	142,165	109,995	100,220	69,225	10	18
			±13,639	±8,246	±8,215	±46,087		
**FB** ^**b**^	12	12/12	138,068	107,926	98,048	55,438	9	49
			±10,917	±10,115	±10,101	±38,385		
**2017–2018**								
**BU** ^**b**^	12	5/12	273,134	117,851	106,113	48,148	10	32
			±55,348	±30,599	±29,132	±68,222		
**SA** ^**b**^	12	8/12	230,022	134,447	123,566	111,187	11	33
			±38,869	±26,489	±21,294	±48,422		
**FB** ^**b**^	12	8/12	237,664	144,687	130,894	119,975	11	30
			±83,593	±64,441	±5,340	±69,933		
**TOTAL**	**84**	**50/84**	**182,722**	**117,996**	**108,000**	**72,685**	**27**	**184**
			**±66,244**	**±33,488**	**±28,751**	**±56,864**		

^a^Shaded values are averages for positive samples.

^b^BU: Bella Unión, SA: Salto, PY: Paysandú, FB: Fray Bentos.

^c^n: number of samples.

^d^pos: positive samples.

^e^OTUs: Operational Taxonomic Units (note that a single EV type could be represented by more than one OTU in a sample).

All 50 positive samples yielded on average 117,996 paired merged reads (contigs), from which around 8.5% reads were discarded according to established quality parameters. Finally, 67.3% (72,685) of filtered contigs mapped with different EVs types (Table 1, S1 Table). In total, 27 different EV types belonging to *Enterovirus A* species (9 types), *Enterovirus B* species (12 types) and *Enterovirus C* species (6 types) were detected (Table 2, S2 Table). Values of nucleotide identity between OTUs and reference sequences with which they were mapped, ranged between 88.2 to 100, 81.1 to 100, and 80 to 98.7 for Enterovirus A, B, and C, respectively (Table 2).

**Table 2:** Distribution of percentage of sequence identity among OTUs and reference sequences with the which they mapped in the Customized Human Enterovirus Database (CHED v1.0).

		Nucleotide identity (%)	
		Min	Max
Enterovirus A	**CVA2**	**88.2**	92.3
	**CVA10**	88.5	97.6
	**CVA3**	88.8	88.8
	**EV-A90**	90.2	90.9
	**CVA5**	92.6	92.6
	**EV-A71**	96,0	98.6
	**CVA4**	96.6	96.9
	**CVA6**	96.9	97.3
	**CVA16**	99.6	**100**
Enterovirus B	**CVB2**	**81.1**	98.4
	**E1**	83.8	83.8
	**E14**	84.3	84.3
	**CVA9**	88.5	94.4
	**CVB5**	91.7	97.8
	**E30**	95.3	96.3
	**CVB4**	96.9	96.9
	**E21**	96.9	97.2
	**E11**	97.8	97.8
	**CVB1**	98.4	98.7
	**E6**	98.4	**100**
	**CVB3**	99.3	99.3
Enterovirus C	**CVA24**	**80.0**	86.1
	**EV-C99**	80.7	**98.7**
	**CVA13**	81.8	86.4
	**CVA1**	86.1	94.7
	**CVA19**	93.9	94.6
	**CVA22**	96.6	98.4

Values in bold are min/max percentages for each Enterovirus species.

Only 5 EV types (CVA10, EV-A71, CVA9, E6 and CVA24) were detected at both sampling periods, while 22 types were detected exclusively in either one of the two sampling periods: 12 types in 2011–2012 (CVA2, A3, A4, A16, A22, B1, B3, E1, E14, E21, EV-A90 and EV-C99), and 10 types in 2017–2018 (CVA1, A5, A6, A13, A19, B2, B4, B5, E11 and E30) (Table 3). Negative samples were not further investigated.

**Table 3:** Enterovirus types detected in wastewater samples from Uruguay in 2011–2012 and 2017–2018.

	2011–2012												2017–2018											
Type^a^/month	**M**	**A**	**M**	**J**	**J**	**A**	**S**	**O**	**N**	**D**	**J**	**F**	**M**	**A**	**M**	**J**	**J**	**A**	**S**	**O**	**N**	**D**	**J**	**F**
CVA2						**X**				**X**														
CVA3											**X**													
CVA4		**X**		**X**																				
CVA5													**X**											
CVA6													**X**										**X**	**X**
CVA10			**X**														**X**	**X**		**X**				
CVA16	**X**	**X**	**X**	**X**	**X**	**X**	**X**	**X**	**X**	**X**	**X**	**X**												
EV-A71											**X**								**X**	**X**				
EV-A90		**X**																						
CVA9			**X**	**X**	**X**		**X**									**X**	**X**							
CVB1							**X**	**X**		**X**	**X**	**X**												
CVB2																**X**	**X**	**X**	**X**	**X**				
CVB3							**X**				**X**	**X**												
CVB4													**X**	**X**										
CVB5														**X**		**X**	**X**			**X**				
E1	**X**																							
E6											**X**		**X**	**X**			**X**		**X**	**X**			**X**	**X**
E11													**X**											
E14				**X**																				
E21	**X**		**X**		**X**																			
E30																**X**	**X**							
CVA1														**X**	**X**	**X**				**X**			**X**	**X**
CVA13																**X**								**X**
CVA19														**X**	**X**	**X**		**X**	**X**	**X**				
CVA22	**X**	**X**	**X**	**X**			**X**			**X**	**X**	**X**												
CVA24			**X**										**X**	**X**	**X**			**X**						
EV-C99	**X**	**X**	**X**	**X**	**X**	**X**	**X**	**X**		**X**	**X**													
EV-C (untyped)			**X** *																					

^a^CVA: coxsackievirus A, CVB: coxsackievirus B, E: echovirus, EV-A: enterovirus A, EV-C: enterovirus C.

* This OTU mapped with reference sequences of different types from Species C (CVA24 and EV-C99). Therefore, it was considered untyped. None of the other 183 OTUs presented incongruent results in the mapping process.

### Enterovirus A71 detection and characterisation

EV-A71 was detected in a sample from January 2012 collected in Paysandú (OTU-C), and in samples from September and October 2017 collected in Bella Unión and Salto, respectively (OTUs A and B); with an abundance (percentage of contigs mapping against EV-A71) between less than 1% and more than 99% per sample (Table 4).

**Table 4:** Defined OTUs of enterovirus A71 and echovirus 30.

	Enterovirus A71			Echovirus 30			
**OTUs** ^**a**^	**OTU-A**	**OTU-B**	**OTU-C**	**OTU-D**	**OTU-F**	**OTU-G**	**OTU-H**
**Location** ^**b**^	**BU**	**SA**	**PY**	**BU**	**BU**	**FB**	**FB**
**Date**	**Sep-2017**	**Oct-2017**	**Jan-2012**	**Jun-2017**	**Jul-2017**	**Jun-2017**	**Jul-2017**
**Abundance (%contigs)**	**99.98%**	**<1%**	**<1%**	**16.40%**	**8%**	**99.97%**	**100%**

^a^OTUs: Operational Taxonomic Units.

^b^BU: Bella Unión, SA: Salto, PY: Paysandú, FB: Fray Bentos.

**Fig 2:**
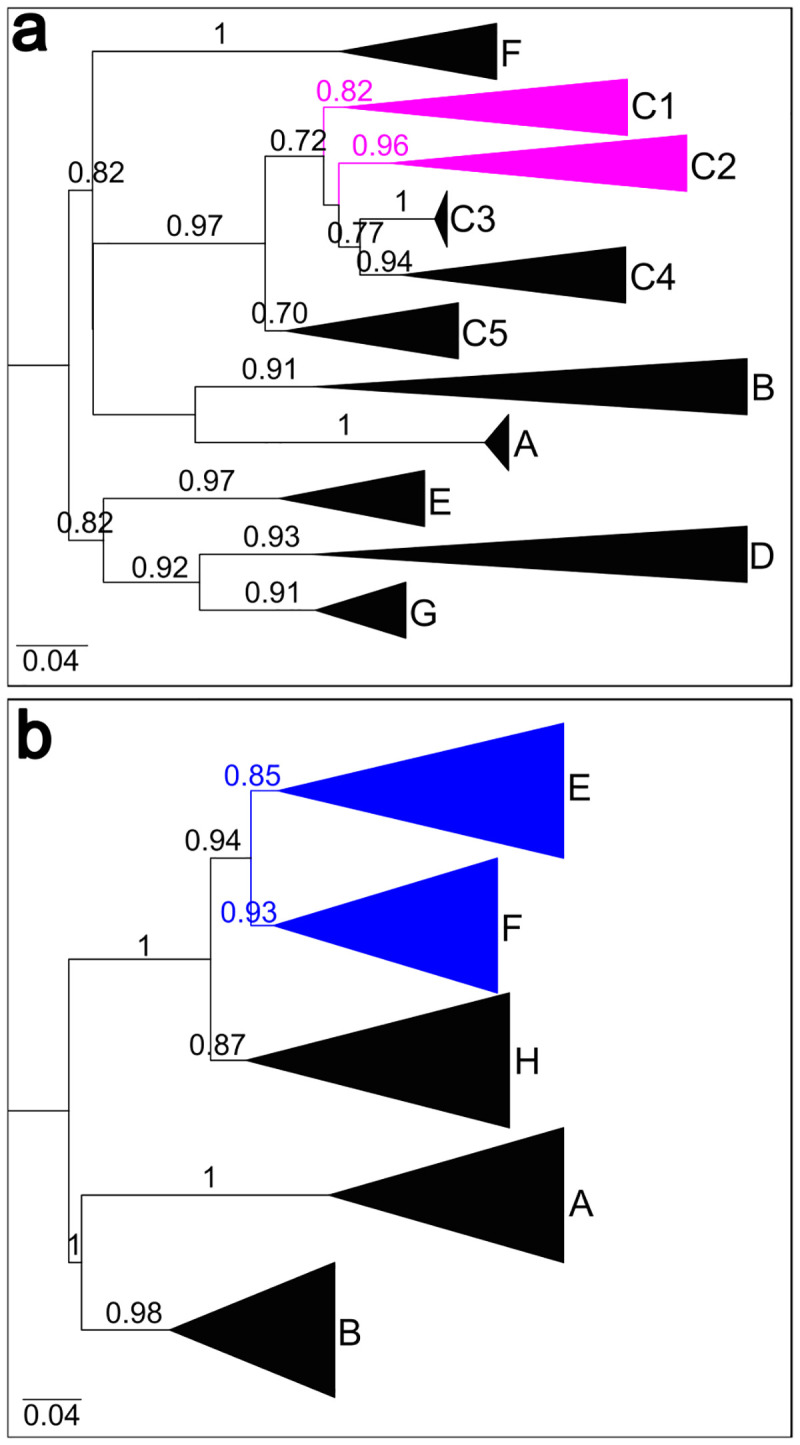
Maximum likelihood phylogenetic trees of enterovirus A71 and echovirus 30. Phylogenetic trees of enterovirus A71 (a) and echovirus 30 (b) were constructed with 7,658 and 1,297 worldwide reported strains, respectively, including 16 strains of EV-A71 and 22 strains of E30 from Uruguay and Argentina reported by us. Clades were collapsed and named according to previously established classification in different lineages. SH-aLRT support values are shown at branches. Each tree is midpoint rooted. For echovirus 30, lineages described by Lema et al. 2019 [35] as extinct were excluded. Bars at the bottom denote genetic distance. Clades containing sequences from this study were highlighted.

These OTUs were subject to phylogenetic analyses along with 7,655 strains worldwide reported. The whole set of sequences segregated into seven different clusters (SH-aLRT ≥ 0.9) according to previously proposed genetic classification of the EV-A71 in genogroups A to G [40]. Additionally, 4,550 strains of genogroup C segregated into sub-genogroups C1 to C5 (SH-aLRT≥0.7) (Fig 2A, S1 File).

**Fig 3:**
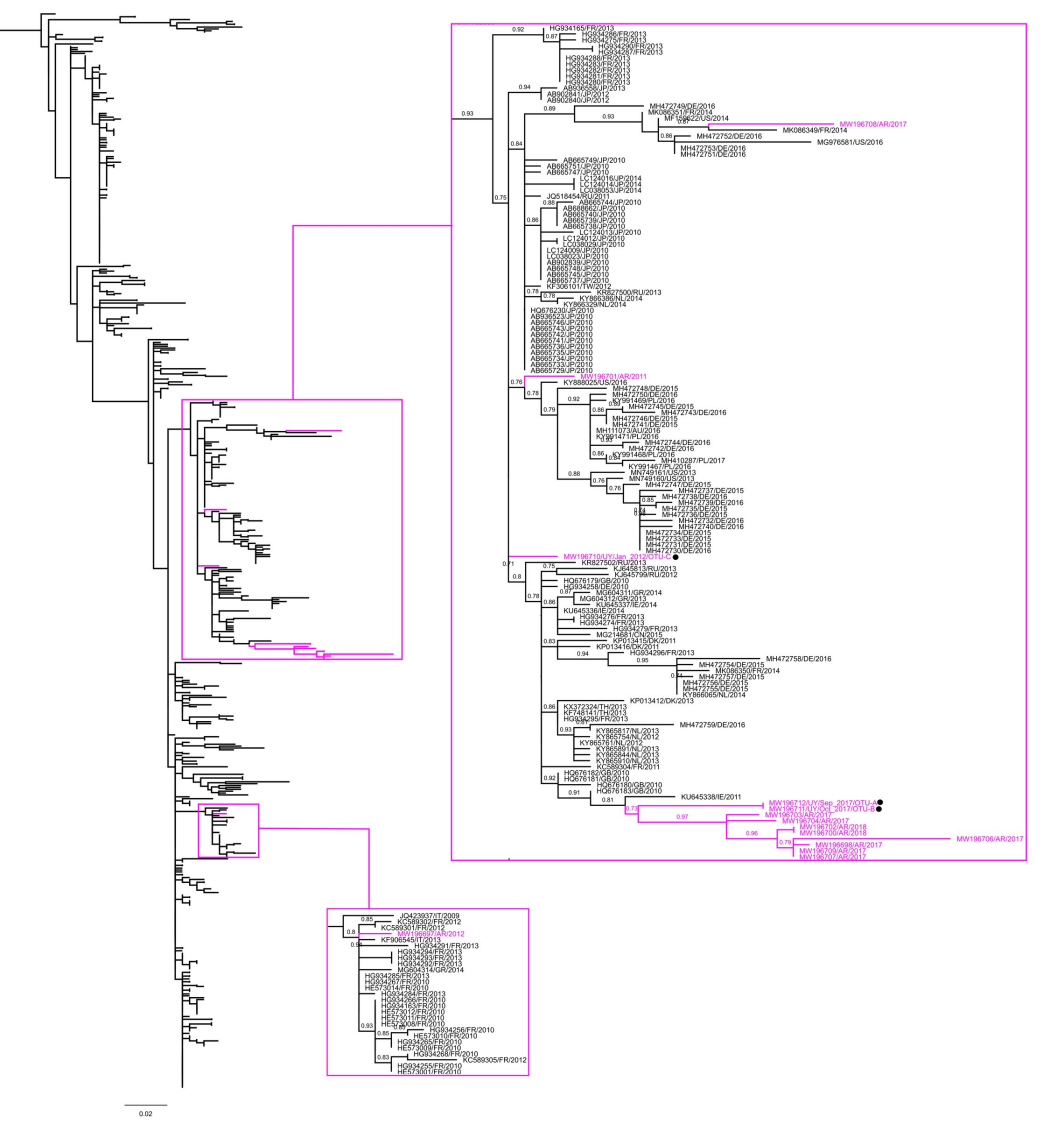
Maximum likelihood phylogenetic tree of enterovirus A71 sub-genogroup C2. The image corresponds to the non-collapsed view of the C2 lineage from Fig 2A, which clustered 573 worldwide circulating strains, including 11 strains from Argentina and 3 strains from Uruguay reported by us in this study. South American strains and clusters that contain them were pink-highlighted, and magnified at right. Strains were named by their GenBank accession numbers, the Alpha-2 country codes (ISO3166), and their detection dates. Black circles highlight Uruguayan strains. SH-aLRT values ≥ 0.7 are shown. The bar at the bottom denotes genetic distance. S3 File shows a full view of the entire tree.

Uruguayan strains of EV-A71 detected in this study clustered together with 559 worldwide strains reported as sub-genogroup C2 and dated between 1995 and 2018 (SH-aLRT = 0.9) (Fig 3, S3 File). Uruguayan OTUs A and B corresponding to Bella Union and Salto respectively, segregated in a monophyletic sub-group (SH-aLRT = 0.73) alongside Argentine strains from 2017 and 2018 previously reported by our group [29]. Phylogenetic analysis suggests that there were at least four other introductions of EV-A71 sub-genogroup C2 into South America (three in Cordoba in 2011, 2012 and 2017, and one in Paysandú in 2012 [OTU-C]) characterised by the absence of local dispersion evidence.

In turn, the sub-genogroup C1 (S4 File) encompassed a sub-cluster composed of two strains from Córdoba Province, Argentina isolated in November and December 2017 alongside 196 sequences from Europe reported in the last 7 years (SH-aLRT = 0.84).

### Echovirus 30 detection and characterisation

E30 was detected in samples from June and July 2017 in Bella Unión and Fray Bentos. Samples from Bella Unión (OTUs-D and F) had less than 20% of contigs mapping with E30, and samples from Fray Bentos (OTUs-G and H) had abundances higher than 99% (Table 4). All E30 OTUs were selected for phylogenetic reconstruction together with 1,293 strains of global circulation including sequences of strains detected previously in wastewater samples from Argentina, as in the case of EV-A71.

**Fig 4:**
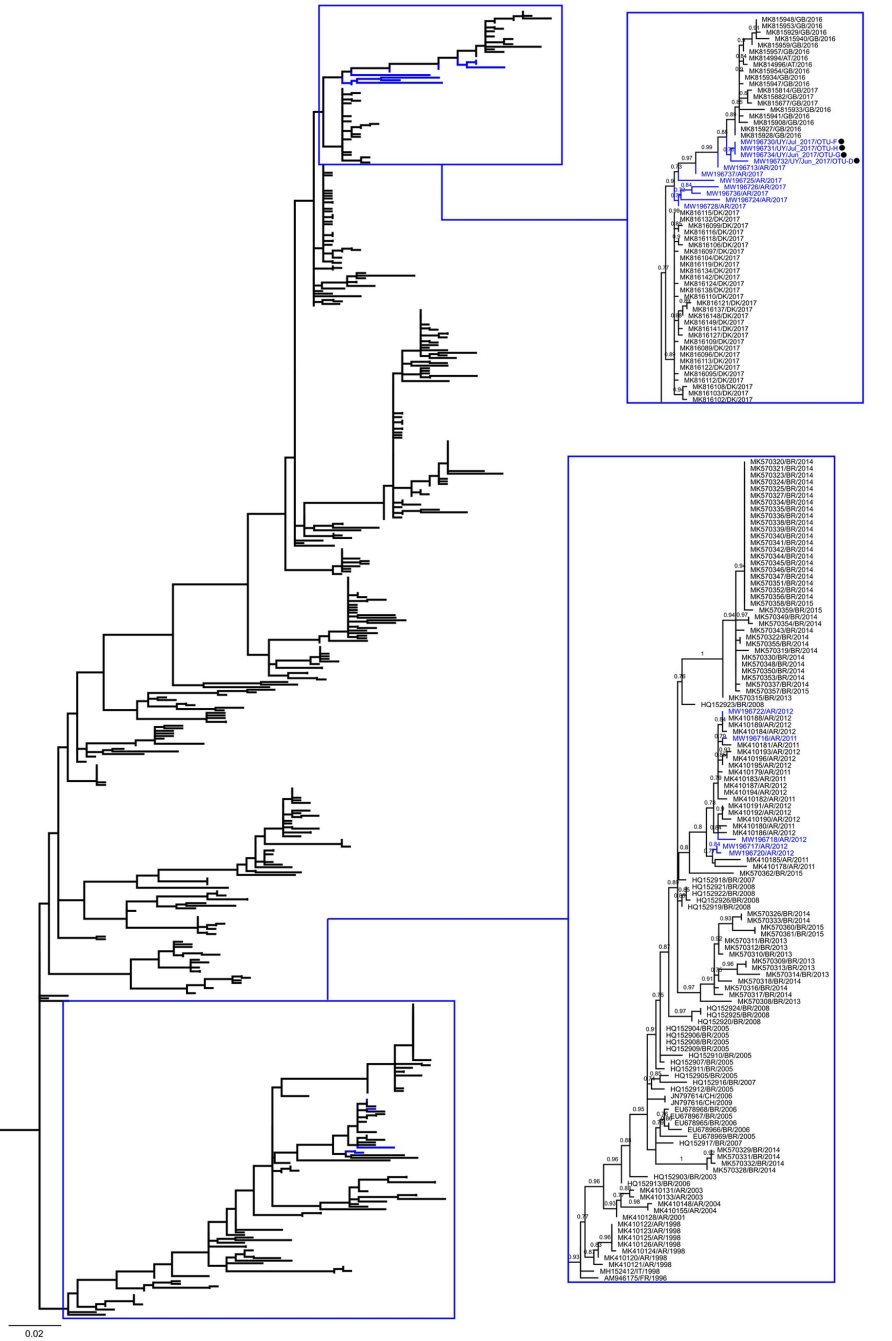
Maximum likelihood phylogenetic tree of echovirus 30 genotype E. The image corresponds to the non-collapsed view of genotype E in Fig 2b, which clustered 508 worldwide circulating strains, including 16 strains from Uruguay and Argentina reported by us in this study (blue branches). Clusters that contain Uruguayan and Argentine strains reported by us are blue-highlighted and magnified at right. Strains were named by their GenBank accession numbers, the Alpha-2 country codes (ISO3166) and their detection dates. Black circles highlight Uruguayan strains. SH-aLRT values ≥ 0.7 are shown. The bar at the bottom denotes genetic distance. S5 File shows a full view of the entire tree.

E30 strains segregated into five well aLRT supported (≥0.85) phylogenetic clusters, representing genotypes A, B, E, F and H after pruning of non-clustered sequences belonging to genotypes C, D and G (Fig 2B, S2 File). The Uruguayan strains of E30 reported in this study segregate into the E genotype alongside sequences from Córdoba-Argentina (all from 2017) previously reported by our group [28] and other 19 strains reported in 2016–2017 in the United Kingdom and Austria (SH-aLRT = 0.9) (Fig 4, S5 File). Additionally, five E30 strains sampled in 2011–2012 in Córdoba and previously reported by our group [29], segregated in a highly supported (SH-aLRT = 0.8) monophyletic group alongside Argentine sequences from the same period reported during an outbreak of AM, and a Brazilian sequence reported in 2015 (Fig 4, S5 File).

On the other hand, six additional E30 strains from Córdoba reported in 2017, clustered together with strains belonging to genotype F (S6 File), and shared a node (SH-aLRT = 0.72) with a sub-cluster of European strains reported between 2014 and 2018.

## Discussion

Twenty-seven different EVs types belonging to *Enterovirus A*, *Enterovirus B* and *Enterovirus* C species were detected in this study, among which 12 circulated in 2011–2012, 10 in 2017–2018, and 5 during both sampling periods.

In a previous study based on Sanger sequencing of amplicons directly obtained from wastewater samples from SET_1 [28], we reported the presence of six out of all types now reported. This means that by this Next-Generation Sequencing approach we expanded our knowledge about the circulation of EVs in Uruguay, and updated the information about their circulation to more recent years.

Contrarily to a previous observation made by us in Córdoba-Argentina [29], where most types detected in 2011–2012 were also identified in samples from 2017–2018, an important type´ exchange was observed between the same sampling periods analysed in Uruguay. A plausible explanation for differences between the results obtained for Cordoba and Uruguay could be related to the population size of the analysed cities and its effect in the duration of transmission chains in the community. We can suppose that in larger populations -Córdoba has 1,329,604 inhabitants- transmission chains are longer than in smaller cities such as those studied by us in Uruguay (the most populated is Salto with 104,028 inhabitants).

Unfortunately, little information is available about the impact of EVs types in the Uruguayan population´ s health and there is a great uncertainty about which EV types are causing AM, encephalitis, or AFP, among other diseases commonly associated with these viruses. Therefore, the implications of the observed exchange of EV types over time for Uruguayan public health, remain unknown.

Since samples from 2011–2012 remained frozen for longer periods than samples from 2017–2018 prior to the sequencing, this could be affecting the diversity of enteroviruses assessed in the oldest samples. Moreover, primers proposed by Nix et al. [30] are quite degenerated and we found some mismatches during the alignment of primers to genomic sequences of enteroviruses that were discovered after the date in the which these primers were designed, which could be affecting the detection of additional types to the reported here. These limitations of our study, lead us to consider that the diversity of enteroviruses in our samples probably could be higher than the described here. Despite the fact that primers designed by Nix et al. are broadly reactive for enteroviruses, future studies should consider more than a single strategy for detecting this group of highly diverse viruses.

EV-A71 is one of the main etiological agents causing HFMD epidemics worldwide, with high incidence in the Asian-Pacific region [40]. There is a strong association between EV-A71 infections and severe forms of HFMD, sometimes with complications at CNS level [41–44]. Interestingly, our results show the *Enterovirus A* species type replacement along the two sampling periods. In 2011–2012, CVA16 circulated predominantly, although in 2017–2018 other types emerged as CVA6 or CVA10, coinciding with reports of HFMD epidemics attributed to these pathogens since 2018 in this geographic region [45, 46]. Nevertheless, EV-A71 was not detected during the study of clinical cases from these epidemics in spite of its detection in wastewater samples collected in Uruguay in September and October, 2017. In the extent that new reports from medical diagnosis describe the presence of EVs in Uruguay, WBE will be an important complement to understand the dynamic of EVs circulation in the country.

Despite global efforts to understand the epidemiology of EV-A71, the knowledge about its circulation in South America is limited. The first genetic record of EV-A71 in South America comes from the detection of genogroup B in Colombia in 1994 [47], and then, in 1999 this genogroup was detected in a stool sample obtained from a patient suffering AFP in Northern Brazil [48]. However, first evidence about the circulation of the virus in Brazil comes from a previous study of serum samples obtained from patients with AFP and paresy [49]. Besides, strains from sub-genogroup C1 were isolated from nasopharyngeal swab samples collected from individuals with flu-like symptoms in Peru in 2006–2009 [50]. Recently, a study found EV-A71 responsible for ~8% of the AFP cases associated with enteroviruses in Brazil in the period 2005–2017 [51]. Interestingly, the study reported that EV-A71 sub-genogroup B1 exclusively circulated up to 2014, and that sub-genogroup C2 replaced it from then. Our study expands the knowledge about the geographic range of sub-genogroup C2 in South America, through the characterization of several strains from both Uruguay and Argentina, representing at least five different introduction events in the region, one of them successfully disseminated in both countries during 2017–2018. Additionally, we evidenced that EV-A71 C2 has circulated in South America at least from 2011, since we detected this sub-genogroup in a sample collected in Córdoba that year.

First evidence about C2 circulation in the Americas are from 1997–1998 and come from the USA [47]. EV-A71 has been characterized in the USA as a virus of low circulation, and specifically C2 was associated with some outbreaks in the middles 2000 [52–54]. However, C2 was detected at a lower frequency than C1 during the study of a large outbreak of neurologic disease in children that occurred in 2018 [55]. Additionally, a low frequency of detection during the study of HFMD cases reported in Cuba between 2011 and 2013, suggests a low circulation of C2 in the Caribbean region too [56].

Unfortunately, since the South American C2 strains herein characterized belong to wastewater samples, and considering the lack of reports from clinical surveillance, it is hard to know whether they come from asymptomatic/mild infections, or from severe diseases such as muscular paralysis or meningitis, that were not reported.

Interestingly, two EV-A71 strains detected in Córdoba in 2017 belong to a sub cluster proposed as an emergent lineage of EV-A71 C1 (C1-like variant), which has been mainly detected since 2015 in European countries [57–62]. The emergence of this C1-like variant in Europe was associated with sporadic cases and outbreaks of severe neurological disorders such as AM, encephalitis or AFP. This meant a concern for countries in which the C1-like variant replaced other sub-genogroups increasing the number of affected individuals, or in where EV-A71 had not been a problem until the C1-like variant entered. Moreover, China has recently reported the emergence of a recombinant strain originated from C1-like variant strains and coxsackievirus A, that could not be effectively neutralized by EV-A71 C4a neutralizing antibodies, which raises concern about the usefulness of the present immunization strategy against the virus [63].

Beyond the first detection of the emergent C1-like variant in South America, our results show the co-circulation of C2 and C1 sub-genogroups in Argentina during 2017, which could propitiate the genomic recombination and upsurge of new variants. Therefore, the epidemic potential of EV-A71 C1-like variant and the possibility of recombination events, together with the severity of the diseases, the long-term sequelae in affected individuals, and the different antigenicity between this variant and vaccine strains, raise concern and call for an active surveillance in our region [63–66].

Although this study constitutes the first report of E30 in Uruguay, this virus was extensively characterised from AM cases in the neighbour countries of Argentina and Brazil [17, 35, 67, 68]. In line with a previous study, we verify the circulation of E30 genotypes E and F in Argentina [35]. The study of AM cases accounted for 2007–2008 in Córdoba [68] found a high predominance of lineage H. Despite this lineage seems to be replaced at local level by E and F in next few years, this data shows how dynamic is the behaviour of E30 in Córdoba, and probably in Argentina all.

In Uruguay, we only detected genotype E. The strains circulating in Uruguay in 2017 were highly related to some strains that circulated in the same year in Argentina, raising the possibility of a single event of introduction -probably from Europe- with a posterior regional dispersion, as was also suggested for EV-A71. On the other hand, some E30 strains detected in wastewater samples from Argentina in 2011–2012, clustered together with other Argentine strains also detected in 2011–2012 during the study of different outbreaks and sporadic cases of AM [35].

Although most of the Argentine E30 strains characterised by our group belong to genotype E, a minor proportion corresponding to samples from Cordoba detected in 2017, belong to genotype F. This genotype circulated in Argentina at least since 1998 when an outbreak of AM occurred in the Province of Mendoza and its last register in the country was in 2007 [35]. In our analysis, strains reported in Brazil in 2017, also clustered inside the genotype F, and strains detected by us in Córdoba in 2017 too, which confirms the recent presence of this genotype in the South American region.

Our results show that a previously unrecognized diversity of EV types circulated in Uruguay. Some of these types silently spread into the communities in the absence of published reports from medical diagnosis about diseases such as AM, encephalitis or AFP. OTUs most divergent regarding our database were mostly from species C (CVA24, EV-C99 and CVA13), which agrees with the previous report of circulation in Uruguay of highly divergent strains from this species [28]. These results emphasise the importance to sequence strains that circulate in remote geographic locations, in order to know divergent strains regarding those already described in the rest of world, as a first step to understand the role of this divergence in the re-emergence of types/subtypes [69]. In line with that, our study is a contribution to elucidate global evolutionary patterns in the extent that divergent members of a genus scarcely described in South America are being characterized.

Specifically, with regards to EV-A71 and E30, our results showed that some variants are widely spread into our region, and others as the emergent EV-A71 C1-like variant -which was detected only in Argentina at the end of 2017- probably entered very recently. Surveillance programs should be strengthened in Uruguay -as well as in other South American countries- to trace the dynamics and behaviour of endemic and emerging variants, especially of those EV types strongly associated with severe diseases poorly studied in our geographic region.

## Supporting information

S1 Table Data processing: Next-generation sequencing results and mapping process. (XLSX)

S2 Table Results of mapping process for all OTUs obtained in this study. (XLSX)

S1 File Maximum likelihood phylogenetic tree (midpoint rooted) constructed with 7,658 partial VP1 fragments of enterovirus A71 strains worldwide reported. The file is optimized for FigTree v1.4.3.
(NXS)

S2 File Maximum likelihood phylogenetic tree (midpoint rooted) constructed with 1,297 partial VP1 fragments of echovirus 30 strains worldwide reported. The file is optimized for FigTree v1.4.3.
(NXS)

S3 File Maximum likelihood phylogenetic tree of enterovirus A71 sub-genogroup C2. (PNG)

S4 File Maximum likelihood phylogenetic tree of enterovirus A71 sub-genogroup C1. The image corresponds to the non-collapsed view of the C1 sub-genogroup in Fig 2a, which clustered 440 worldwide circulating strains, including 2 strains from Argentina reported by us (blue branches). SH-aLRT ≥0.7 are shown at branches. The C1 like-variant sub-cluster is highlighted in red. GenBank accession numbers, Alpha-2 country codes (ISO3166) and detection dates code each strain. The bar at the bottom denotes genetic distance.
(PNG)

S5 File Maximum likelihood phylogenetic tree of echovirus 30 genotype E. (PNG)

S6 File Maximum likelihood phylogenetic tree of echovirus 30 genotype F. The image corresponds to the non-collapsed view of the F genotype in Fig 2b, which clustered 350 worldwide circulating strains, including 6 strains from Argentina (blue branches) reported by us. SH-aLRT ≥0.7 are shown at branches. GenBank accession numbers, Alpha-2 country codes (ISO3166) and detection dates code each strain. The bar at the bottom denotes genetic distance.
(PNG)
